# The surgeon and his tools-the case for a focused orthopaedic theatre induction programme

**DOI:** 10.1186/1756-0500-1-104

**Published:** 2008-10-28

**Authors:** AJ Shyam Kumar, J Oakley, Shaun KS Wong, Steve J Philips

**Affiliations:** 1 Specialist registrar in Trauma & Orthopaedics, Wrexham Maelor hospital, Wrexham, UK; 2 Senior house officer in Orthopaedics, Ysbyty Gwynedd, Bangor, UK; 3 Consultant in Trauma & Orthopaedics, Wrexham Maelor hospital, Wrexham, UK

## Abstract

**Background:**

Induction programme for trainee doctors in the UK generally do not focus on the surgical aspects of their jobs. In this context we decided to conduct a telephonic survey among the hospitals belonging to three orthopaedic training regions in the UK from the point of view of the diversity of instrumentations and implants used for index procedures.

**Results:**

We chose four index trauma & orthopaedic procedures (Total hip replacement, total knee replacement, intramedullary nailing and external fixator systems for long bone fractures). A telephonic survey was done in six NHS trust hospitals which were part of an orthopaedic training rotation (2 from England, 2 from Wales and 2 from Scotland). In total there were 39 different instrumentation systems for these 4 index procedures in the 6 trusts (see table 1). These comprise 12 Total hip replacement (THR) systems, 14 total knee replacement (TKR) systems, 9 intra-medullary nailing systems, and 4 external fixator systems. The number of different systems for each trust ranged from 7 to 19. There is a vast array of implants and instrumentation systems in each trust, as highlighted by our survey. The surgical tools are not the same in each hospitals. This situation is more complicated when trainees move to new hospitals as part of training rotations.

**Conclusion:**

In view of this we feel that more focused theatre based induction programmes for higher surgical trainees is advocated in each hospital trust so trainees can familiarise themselves with the tools available to them. This could include discussion with the consultants and senior theatre staff along with representatives from the companies supplying the implants and instrumentation systems.

**Table 1:** Number of implants/instrumentations used in each of the 6 UK trusts (3 training regions).

IMPLANT	E1	E2	W1	W2	S1	S2
*Total Knee Replacement*	4	5	2	4	3	2
*Total Hip Replacement*	3	4	3	6	3	3
*Intramnedullary nailing*	2	1	1	6	2	3
*External fixators*	2	3	2	2	1	1

*TOTAL*	11	13	8	18	9	9

E = England, W = Wales, S = Scotland

## Background

Hospital induction programmes are a standard part of the commencement of new posts for junior doctors [1]. It has been reported that the content and style of induction programmes vary greatly between hospitals [2] and tend to target more junior doctors such as foundation years one and two (FY1, FY2) [3-6]. From our experience, as higher surgical trainees (past and present) on specialist orthopaedic training programmes, we feel there is a need for more emphasis on a focused approach to specific departmental induction programmes. In particular, induction to the implants and equipment utilised in each particular NHS trust. The practice of trauma and orthopaedic surgery involves the use of a vast array of instrumentation and implants. For clinical decision making and management, we as surgeons must be aware of the various tools at our disposal and should be familiar with them. This is of particular importance in emergency situations. As far as we are aware there are no structured orthopaedic instrumentation induction programmes for specialist registrars in the United Kingdom. As a result when we move from one hospital to the other we are suddenly faced with unfamiliar instrumentation systems and implants. This therefore, increases the learning curve for many elective and trauma procedures that we must master as trainees.

## Methods

To emphasise the scale of this problem a telephonic survey was done in six NHS trust hospitals which were part of an orthopaedic training rotation (2 from England, 2 from Wales and 2 from Scotland). In our survey we used 4 index operations commonly performed in all hospitals with a trauma and orthopaedic intake. For each trust we contacted (by telephone) the theatre manager to identify the instrumentation and implant systems used for total hip replacement (THR), total knee replacement (TKR), and fixation of tibial or femoral fractures with either intra-medullary nailing or external fixation.

## Results

In total there were 39 different instrumentation systems for these 4 index procedures in the 6 trusts (see table 1). These comprise 12 THR systems, 14 TKR systems, 9 intra-medullary nailing systems, and 4 external fixator systems. The number of different systems for each trust ranged from 7 to 19.

## Discussion

This representative survey we conducted shows no uniformity in the orthopaedic instrumentation used between the different regions. This is hardly surprising as currently there is a vast array of instrumentation systems available in the market for use in orthopaedic surgery. Considering that this data only represents 4 particular index operative procedures the actual amount of different implants and instrumentation systems for each trust is likely to be significantly higher.

We recommend mandatory theatre induction as a part of the specialist registrars' departmental induction programme. This could include discussion with the consultants and senior theatre staff along with representatives from the companies supplying the implants and instrumentation systems. Ideally it would include practical/video demonstrations of the commonest systems used, as well as a booklet for each trainee containing the operative technique for the instrumentation systems.

## Competing interests

The authors declare that they have no competing interests.

## Authors' contributions

AJSK designed the survey and wrote the article, JO and SKSW conducted the survey and the literature search, SP revised the manuscript and gave final approval for publication
